# Genetics & Epigenetics of Hereditary Deafness: An Historical Overview

**DOI:** 10.3390/audiolres11040057

**Published:** 2021-11-17

**Authors:** Alessandro Martini, Flavia Sorrentino, Ugo Sorrentino, Matteo Cassina

**Affiliations:** 1 Padova University Research Center “International Auditory Processing Project in Venice (I-APPROVE)”, “Santi Giovanni e Paolo” Hospital, 30122 Venice, Italy; 2 Otolaryngology Unit, Department of Neurosciences, University of Padova, 35128 Padova, Italy; flavia.sorrentino@unipd.it; 3 Clinical Genetics Unit, Department of Women’s and Children’s Health, University of Padova, 35128 Padova, Italy; ugo.sorrentino@aopd.veneto.it

**Keywords:** genetics, epigenetics, hearing loss, deafness, NGS

## Abstract

Hearing loss (HL) is one of the most common sensory impairments worldwide and represents a critical medical and public health issue. Since the mid-1900s, great efforts have been aimed at understanding the etiology of both syndromic and non-syndromic HL and identifying correlations with specific audiological phenotypes. The extraordinary discoveries in the field of molecular genetics during the last three decades have contributed substantially to the current knowledge. Next-generation sequencing technologies have dramatically increased the diagnostic rate for genetic HL, enabling the detection of novel variants in known deafness-related genes and the discovery of new genes implicated in hearing disease. Overall, genetic factors account for at least 40% of the cases with HL, but a portion of affected patients still lack a definite molecular diagnosis. Important steps forward have been made, but many aspects still have to be clarified. In particular, the role of epigenetics in the development, function and pathology of hearing is a research field that still needs to be explored. This research is extremely challenging due to the time- and tissue-dependent variability of the epigenetic changes. Multisystem diseases are expected to be investigated at first: specific epi-signatures have been identified for several syndromic disorders and represent potential markers for molecular diagnostics.

## 1. Introduction

Hearing loss (HL) is one of the most common sensory impairments worldwide and represents a critical medical and public health issue [1]. Etiologies vary according to the age at onset of HL and include genetic, infective, toxic and environmental factors. Overall, genetic factors account for at least 40% of the cases, and a relevant portion of affected patients can have a definite molecular diagnosis thanks to Next Generation Sequencing (NGS) technologies [1,2,3].

Since the mid-1900s, great efforts have been aimed at understanding the causes of both syndromic and non-syndromic HL and identifying correlations between specific etiological factors and the associated audiological phenotype.

## 2. Genetics of Deafness: From Linkage Analyses to Next Generation Sequencing

In 1955, Fisch demonstrated a correspondence between audiometric patterns and the etiology of perceptive deafness. He wrote that “*a flat audiogram suggests rubella, a saucer-shaped audiogram kernicterus, a gently sloping audiogram with the high tones affected more than the low is often seen in dominant deafness and a sharply sloping audiogram with a residual island of hearing in the low tones suggests autosomal recessive deafness*” [4]. In 1970, Fraser reported on a large sample of 3534 individuals who had been *“profoundly deaf from childhood*”, describing many syndromic cases, and paying particular attention to the causes based “*on family history*” [5]. In 1971, Walter Nance published “Genetic Counselling for the Hearing Impaired” [6]; in addition, in the same year, Bruce W. Koniksmark titled his contribution to “The second Conference on The Clinical Delineation of Birth Defects”, Syndromal Approaches to the Nosology of Hereditary Deafness [7]. He wrote: “*There are about 70 types of hereditary deafness in man. The differential diagnosis of these familial deafness syndromes is aided by using the following five characteristics of the syndrome: (1) the mode of genetic transmission, (2) the characteristics of the deafness, (3) the age of onset, (4) the sonic frequencies involved and (5) the associated abnormalities*”. Bruce Konigsmark’s fundamental book (completed by Robert Gorlin after Bruce’s premature death in 1973), “Genetic and Metabolic Deafness”, was published in 1976 [8]. This book and subsequent editions of it [9,10], as well as McKusick’s “Mendelian Inheritance in Man” and its online version [11,12,13], have been important reference sources for clinicians for decades.

The first loci associated with HL were mapped by linkage analysis within large and clinically well-characterized families; moreover, homozygosity mapping was another approach used to identify autosomal recessive loci in consanguineous families. The first non-syndromic HL locus was mapped in 1988 by studying families showing X-linked inheritance (DFNX2) [14,15]; seven years later, *POU3F4* (MIM *300039) was identified as the gene associated with the phenotype [16]. The first autosomal dominant locus (DFNA1) was linked to chromosome 5q31 in 1992 [17], leading to the identification of *DIAPH1* gene (MIM *602121) few years later [18]. The first autosomal recessive locus (DFNB1) was mapped in 1994 [19], and pathogenic variants in *GJB2* gene (MIM *121011) encoding Connexin 26 were identified in 1997 [20].

An important problem during early years was the lack of standardization of terms, especially those regarding the auditory phenotype; patients’ clinical description was often unprecise or incomplete in papers from the medical genetics literature, making further analyses and genotype–phenotype correlations challenging. A great impact in this field was given by the establishment of international research groups allowing the collaboration between Clinical Otologists/Audiologists and Geneticists, starting from HEAR (Hereditary deafness Epidemiology And clinical Research) in 1994 and GenDeaf (European thematic network on genetic deafness) in the 2000s [21,22,23,24,25].

The impressive discoveries in the field of molecular genetics during the last three decades have contributed substantially to the current knowledge on the etiology and pathology of several forms of hearing impairment. Mutations located in hundreds of genes have been identified as causative factors of syndromic and non-syndromic HL. To date, about 161 non-syndromic HL loci (66 autosomal dominant loci, 88 autosomal recessive loci, six X-linked loci and one Y-linked locus) have been reported, and 122 non-syndromic HL-associated genes have been identified (Hereditary Hearing Loss Homepage; https://hereditaryhearingloss.org/ accessed on 14 September 2021) [26]; in addition, more than 400 syndromes with HL included in their phenotypic spectrum have been described [13].

The introduction of next-generation sequencing (NGS) technologies, such as targeted resequencing and whole exome sequencing (WES), has dramatically increased the diagnostic rate for HL, enabling the detection of novel variants in known deafness-related genes and the discovery of new genes implicated in hearing disease [27]. The problem now concerns the interpretation of all these sequencing data and the correct classification of the detected variants. Richard Smith and co-authors [28] reported that “*the classification of genetic variants represents a major challenge in the post-genome era by virtue of their extraordinary number and the complexities associated with ascribing a clinical impact, especially for disorders exhibiting exceptional phenotypic, genetic and allelic heterogeneity*”. The classification of variants has to take into account several criteria, as proposed by the recommendations of the American College of Medical Genetics and Genomics; these include the type of the variation, the allele frequencies in population databases aggregating data from sequencing projects, the pathogenicity predictions of in silico algorithms, the identification of the variant in other cases with overlapping phenotype in the literature and the availability of in vitro or in vivo functional studies [29]. In addition, it is important to note that genetic variations classified as pathogenic in the pre-NGS era literature, especially those reported without functional studies proving their deleterious effect, need to be reconsidered now. The data obtained from genome and exome sequencing projects have allowed to better understand the human genetic variability and the frequencies of specific variants in different populations; with this essential information, several mutations have been re-classified as variants of unknown significance (VUS) or as benign variants. Several updated databases on variant classification are available and represent invaluable resources for diagnostic laboratories personnel and clinicians. Among these, the Deafness Variation Database (https://deafnessvariationdatabase.com/) is curated by experts in the field of HL diagnosis and research and is freely available to the public; it includes annotation and classification of thousands of genetic variants related to syndromic and non-syndromic HL.

Significant advances in the field of HL genetics are expected from Whole Genome Sequencing (WGS) [30]. This analysis allows the detection of genetic variants in both coding and non-coding regions and the identification of structural variations. However, several limitations still prevent WGS application in clinical diagnostics: it is more expensive than the other NGS-based analyses (targeted resequencing of gene panels and WES) and provides a huge volume of data that needs to be processed and stored. Moreover, although the knowledge in genomics is constantly growing, the interpretation of variants in non-coding regions is a big issue, and functional studies are usually necessary to demonstrate pathogenicity. Therefore, WGS is now used only in selected cases, mainly within research projects [30]. Finally, the development of third generation/long-read sequencing technologies will also improve the detection rate of structural variations and the discrimination of regions with high sequence homology [31].

Exploring the genetic landscape of HL is a crucial step to comprehend the biological pathways involved in the pathogenesis of this sensory impairment and to identify specific genotype–phenotype correlations. The currently available NGS-based tests are extremely powerful tools that allow an accurate determination of the etiology of HL in a large proportion of affected patients and may provide important prognostic information, including potential medical complications and guidance on long-term medical management. Furthermore, a definite molecular diagnosis is fundamental to know the recurrence risk of the disorder and to provide accurate genetic counseling.

## 3. Future Perspectives: Epigenetics of Hearing Loss

Important steps forward have been made in the understanding of the molecular bases of HL, but many aspects still have to be clarified. In particular, the role of epigenetics in the development, function and pathology of hearing is a research field that needs to be explored.

Advances in genomic technologies have enabled a comprehensive look at the key mechanisms of epigenetics, and their understanding has become a major focus for research in most biological systems. However, while thousands of papers have been published on epigenetics in general and on its involvement in embryogenesis, development and on the pathogenesis of cancer and many other human disorders, very few studies have been focused on epigenetics and hearing.

The term “epigenetics” was coined in 1942 by the embryologist and developmental biologist Conrad Waddington to define a “*branch of biology that studies the causal interactions between genes and their products which bring the phenotype into being*” [32,33]. In 1958, Nanney [34], five years after James Watson and Francis Crick first published the 3D structure of the DNA double helix [35], published a paper in which he used the term epigenetics to distinguish between different types of cellular control systems, trying to explain the relationships between genotype and phenotype [36]. He wrote in PNAS: “*Alterations in the genetic material are thought to come about in one of two major fashions. Mutations are the more or less random alterations in the code which result from chance substitutions in the nucleotide sequences or from gains or losses of nucleotides. Recombination also results in changes in the code, but these changes are achieved in a more orderly fashion…; Difficulties arise, however, when one attempts to determine whether observed differences in cellular properties are due to differences in the “primary genetic material” or to differences in other cellular constituents … To simplify the discussion of these two types of systems, they will be referred to as “genetic systems” and “epigenetic systems.” The term “epigenetic” is chosen to emphasize the reliance of these systems on the genetic systems and to underscore their significance in developmental processes*”. [34,37].

Epigenetics is a field of study that focuses on alterations in the expression of genes, rather than changes of the gene sequence itself, and how that affects phenotype; it was also described as “the science of change” [38]. Examples of epigenetic mechanisms involved in gene expression and the related phenotype determination are DNA methylation, histone modifications and non-coding RNAs [39,40]; these mechanisms play an essential role in the development from a single totipotent zygote through defined subsets of multipotent progenitor cells, eventually yielding the entire variety of cell types in an adult organism [41].

Epigenetic dysregulations and modifications have been associated with many human disorders and can be induced and modulated by the exposures to environmental factors (such as nutrients, pollution, toxicants and inflammation), both prenatal and post-natal. It is now known that epigenetic modifications may also persist via meiosis and thus be heritable.

Moreover, epigenetics is a leading actor in the new “gender” and “precision” medicine to explain the modifiers of the most common causes of death, morbidity and therapeutical responsiveness, and it articulates the genetic, biological and environmental determinants that underlie these differences [42,43].

The important role of epigenetics in ear development and functioning has been suggested by several human genetic disorders caused by mutation in genes coding for factors involved in DNA methylation, histone modification and chromatin remodeling; ear defects and HL are included in the phenotypic spectrum of several of these conditions. Furthermore, it is tempting to speculate that epigenetic alterations themselves may play a primary role in hearing-related diseases and syndromes without clear pathogenic variations of the DNA sequence [40,44].

In the field of HL related to craniofacial malformation, Riccardi was probably the first scientist to consider the role of epigenetics in the determination of the variable expressivity of genetic disorders; in his presentation, “Cell–cell interaction as an epigenetic determinant in the expression of mutant neural crest cells”, at the Symposium on “Developmental Aspects of Craniofacial Dysmorphology” held in San Francisco in 1978 [45], he wrote: “*Recent clinical investigation of patients and families with von Recklinghausen neurofibromatosis suggest that another type of explanation may account for variable expression of at least some autosomal dominant mutations. This alternative explanation relies on factors that are extrinsic to the mutant NFT gene and can be viewed as an epigenetic model*”.

The role of epigenetic factors in phenotype expression is particularly evident in monozygotic twins; in fact, they are rarely completely identical despite their origin from a single zygote. In 1997, Elçioğlu and Berry [46] reported on discordant monozygotic twin girls, one of whom was diagnosed as having IVIC (Oculo-oto-radial) syndrome based on hand abnormalities and HL, but her twin sister had only strabismus. The family had at least seven apparently and two possibly affected members over four generations, the majority being only mildly affected. The two girls showed marked phenotype variability, suggesting that modification of expression must be epigenetic or environmental, rather than genetic.

Phenotype variability is now a well-known characteristic of many genetic disorders, especially autosomal dominant diseases, even in patients carrying the same genetic alteration. Phenotypic features that show variable expression must be influenced by several factors including epigenetic modifications, among others (genetic background, somatic mutations and environmental factors).

The study of epigenetic modifications associated with HL is extremely challenging, especially in case of non-syndromic conditions. In fact, epigenetic changes vary in the different tissues of the same individual, and modifications detected in blood cells or other tissues may not reflect those present in the ear. Therefore, the study of non-syndromic HL would benefit from the collection of tissue samples of the inner ear, which is a difficult procedure in the research setting [47]. In addition, the study of epigenetic modifications involved in the development of the inner ear is complex, relying also on the analysis of fetal samples collected at different developmental stages [48]. Therefore, the first studies are focusing on syndromic disorders caused by pathogenic variants in epigenetic regulatory genes. Characteristic and overlapping methylation signatures (epi-signatures) in peripheral blood DNA have been observed in several disorders resulting from defects in various layers of the epigenetic machinery, including those involving abnormalities in chromatin remodeling [49]. These epi-signatures have been used as predictive tools for the classification of variants of uncertain significance as pathogenic or benign and could be potentially used in the clinical setting as markers for molecular diagnostics, with performances even superior to sequence variant analysis [49,50].

In conclusion, epigenetics is involved in important physiologic and pathologic processes of ear development and function [41,47]; the understanding of the underlying mechanisms is challenging but may represent an enormous promise for preventing and treating HL in humans [39].

## Data Availability

Not applicable.
